# Asymptomatic Colonic Diverticulosis With Sigmoid Colon Perforation and Barium Peritonitis After an Upper Gastrointestinal Series: A Case Report

**DOI:** 10.7759/cureus.110484

**Published:** 2026-06-08

**Authors:** Yusuke Makutani, Shumpei Satoi, Yoshifumi Takeyama

**Affiliations:** 1 Department of Surgery, Osaka Gyoumeikan Hospital, Osaka, JPN

**Keywords:** asymptomatic diverticulosis, barium peritonitis, colonic diverticulosis, diverticular perforation, upper gastrointestinal series

## Abstract

Upper gastrointestinal series (UGIS) remains an important modality for gastric cancer screening; however, intestinal perforation after this examination, although rare, can result in life-threatening barium peritonitis. We report a case of sigmoid colon perforation after UGIS in a 47-year-old woman with previously asymptomatic colonic diverticulosis and discuss its implications for preexamination risk assessment. She developed abdominal pain two days after undergoing UGIS during a routine health checkup. Computed tomography showed free air and intraperitoneal barium leakage. Emergency surgery revealed sigmoid colon perforation with a barium fecalith adjacent to the perforation site. Partial sigmoid colectomy, diverting transverse colostomy, extensive peritoneal lavage, and drainage were performed. Postoperatively, she developed septic shock and required intensive care and continuous hemodiafiltration; however, she gradually recovered and was discharged on postoperative day 40. Histopathological examination suggested diverticular perforation without malignancy. In this case, asymptomatic colonic diverticulosis may have reduced colonic wall compliance, and localized pressure from the barium fecalith may have triggered perforation. Given the increasing prevalence of colonic diverticulosis, preexamination risk assessment before UGIS should consider asymptomatic diverticulosis as well as treated diverticulitis.

## Introduction

Upper gastrointestinal series (UGIS) is widely used as one of the modalities for gastric cancer screening and remains clinically important [1]. UGIS has also been reported to be more useful than endoscopy for diagnosing and localizing scirrhous gastric cancer [2]. Although the use of barium studies has declined in many Western countries with the widespread availability of endoscopy and cross-sectional imaging, they remain useful in selected clinical settings [3,4]. Therefore, UGIS continues to have clinical significance.

However, intestinal perforation after UGIS, although rare, can lead to a severe clinical course because of barium peritonitis [5,6]. The mortality rate has been reported to range from 20% to 50% [7,8]. The underlying pathophysiology is thought to involve massive exudation of fluid and protein caused by the rapid spread of leaked barium throughout the peritoneal cavity, sepsis associated with fecal contamination, and impairment of intraperitoneal defense mechanisms caused by barium itself [9,10].

To avoid such serious complications, contraindications and exclusion criteria for gastric barium X-ray examinations have been established in Japan, and one such criterion is a history of treated diverticulitis [11]. However, asymptomatic colonic diverticulosis is not included in the current exclusion criteria.

In this report, we describe a case of colonic perforation with barium peritonitis after an upper gastrointestinal barium examination in a patient with asymptomatic colonic diverticulosis. In addition, based on a review of the literature, we discuss pre-UGIS risk assessment in light of the current knowledge regarding asymptomatic colonic diverticulosis.

## Case presentation

A 47-year-old woman underwent an upper gastrointestinal barium X-ray examination as part of a routine health checkup. Following the examination, she was prescribed sennoside 12-mg tablets at a dose of six tablets daily in three divided doses for one day and was instructed to maintain adequate oral hydration. She developed abdominal pain two days after the examination and was admitted to our hospital. Her medical history included congenital left renal atrophy, renal dysfunction, and chronic constipation. She had no documented history of colonic diverticulosis. Her family history was unremarkable.

On admission, she had diffuse abdominal tenderness with rebound tenderness. Laboratory examination showed no marked inflammatory response. Laboratory findings at admission are summarized in Table 1. Noncontrast abdominal computed tomography (CT) revealed free air and intraperitoneal leakage of barium (Figure 1).

**Table 1:** Laboratory findings at admission CRP: C-reactive protein; BUN: blood urea nitrogen

Parameter	Patient value	Reference range
White blood cells	6,650/µL	3,500-9,100/µL
Hemoglobin	10.1 g/dL	11.3-15.2 g/dL
Platelet	25.9 × 10^4^/µL	13-36.9 × 10^4^/µL
Albumin	4.0 g/dL	3.7-5.2 g/dL
CRP	0.13 mg/dL	0-0.3 mg/dL
Creatinine	2.07 mg/dL	0.41-0.76 mg/dL
BUN	30.5 mg/dL	8-22 mg/dL

**Figure 1:**
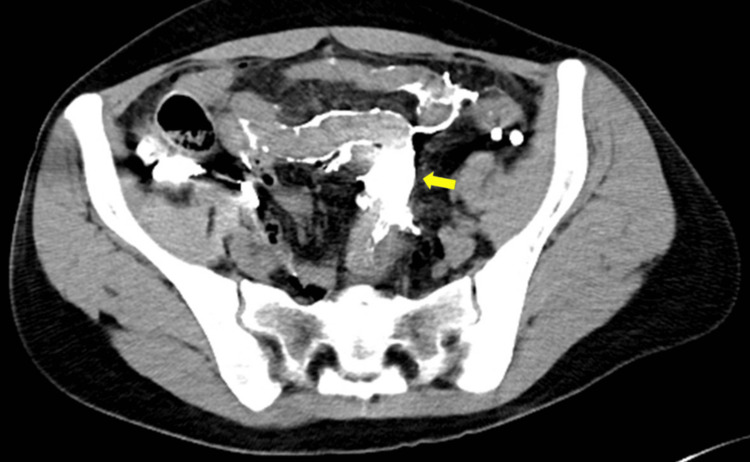
Noncontrast abdominal computed tomography showing intraperitoneal barium leakage (yellow arrow) with free intraperitoneal air

Based on these findings, gastrointestinal perforation was diagnosed, and emergency surgery was performed on the same day. Intraoperatively, feculent contamination was observed throughout the abdominal cavity, predominantly in the left lower quadrant. A perforation was identified in the sigmoid colon (Figure 2), and a barium fecalith was found adjacent to the perforation site (Figure 3). Although severe intra-abdominal contamination was observed, inflammation in the remaining bowel was relatively mild. There was no marked bowel wall edema or apparent mucosal injury, and the bowel tissue was judged to be suitable for anastomosis. Therefore, segmental resection of the sigmoid colon, including the perforation site, was performed, followed by reconstruction using a functional end-to-end anastomosis.

**Figure 2:**
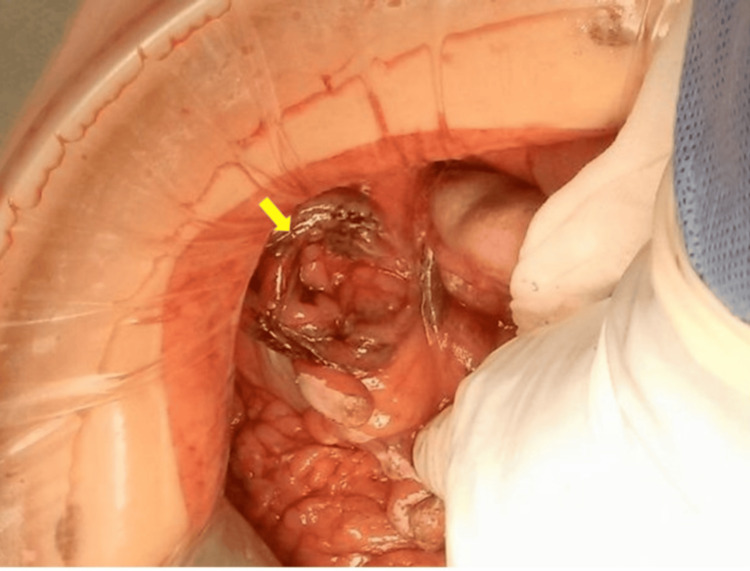
Intraoperative findings showing a perforation in the sigmoid colon (yellow arrow)

**Figure 3:**
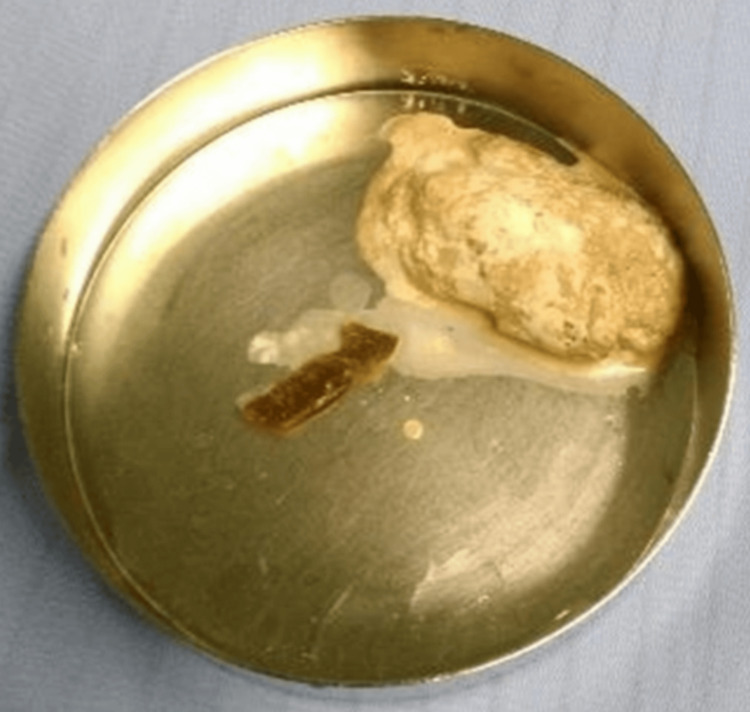
Barium fecalith found in the abdominal cavity, measuring 5 cm in diameter

In addition, because preoperative CT demonstrated a large amount of retained stool and barium in the transverse colon (Figure 4), a double-barrel transverse colostomy was created to divert fecal flow and reduce the clinical impact of potential anastomotic leakage. The abdominal cavity was thoroughly irrigated with more than 10 L of normal saline, and drains were placed in the left subphrenic space, right subphrenic space, and Douglas’ pouch before completion of the operation.

**Figure 4:**
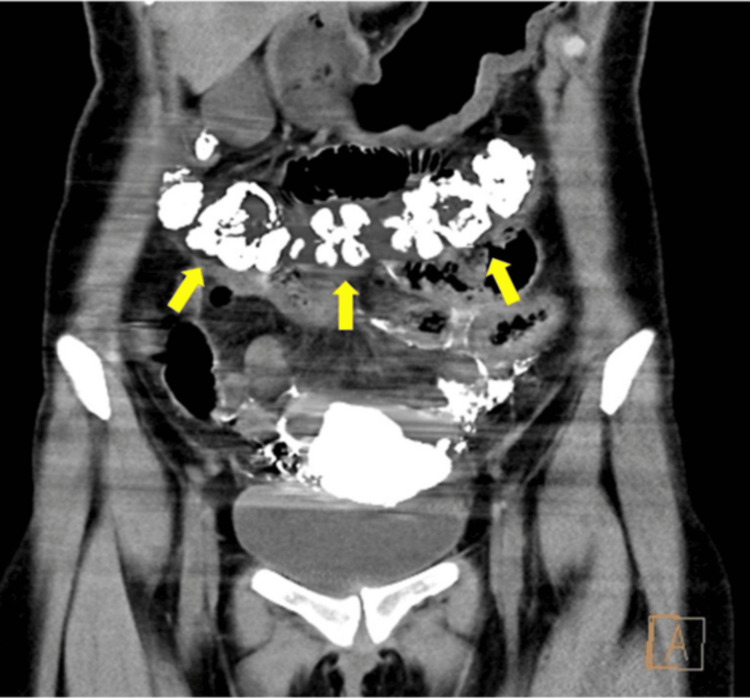
Noncontrast abdominal computed tomography showing retention of feces and barium in the transverse colon (yellow arrow)

Because the patient developed septic shock and required high-dose vasopressor support, she remained intubated after surgery and was admitted to a high-dependency unit. Her septic shock was complicated by disseminated intravascular coagulation, and her renal dysfunction worsened; therefore, continuous hemodiafiltration (CHDF) was initiated on postoperative day 3. Her general condition remained unstable postoperatively, but the endotoxin level measured on postoperative day 4 was within the normal range. Thereafter, her hemodynamic status gradually stabilized, and she was extubated on postoperative day 10. CHDF was switched to hemodialysis (HD) on postoperative day 15, and she was discharged from the high-dependency unit on postoperative day 16. Her urine output gradually increased thereafter, and HD was discontinued on postoperative day 33. After rehabilitation, she was discharged home on postoperative day 40.

Postoperative laboratory tests showed persistently elevated inflammatory markers (Figure 5); however, blood cultures obtained on postoperative days 9, 19, and 32 were all negative. In addition, CT performed on postoperative days 11 and 22 demonstrated barium distributed along the intestinal wall (Figure 6), but no obvious infectious source, such as pneumonia, intra-abdominal abscess, subcutaneous abscess, or anastomotic leakage, was identified. Macroscopic examination of the resected specimen revealed multiple diverticula (Figure 7). Histopathological examination showed invagination of the colonic mucosal epithelium into the subserosal layer at the perforation site, accompanied by inflammatory cell infiltration predominantly composed of neutrophils. These findings were considered compatible with perforation associated with a diverticulum. No abscess formation, ischemic changes, or ulcer formation suggestive of stercoral-type ulceration were identified. No evidence of malignancy was observed.

**Figure 5:**
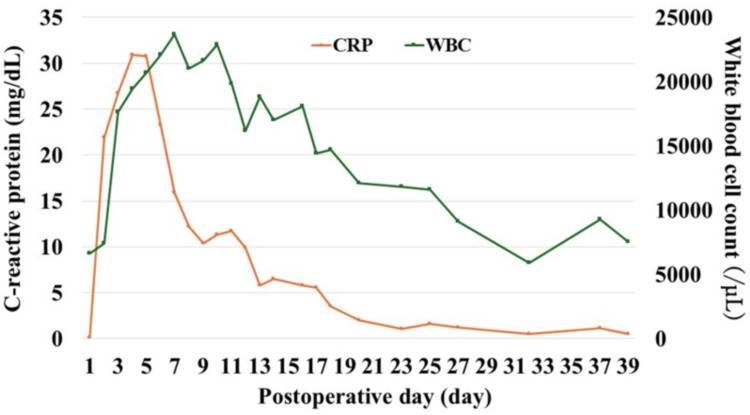
Postoperative changes in serum C-reactive protein levels and white blood cell counts

**Figure 6:**
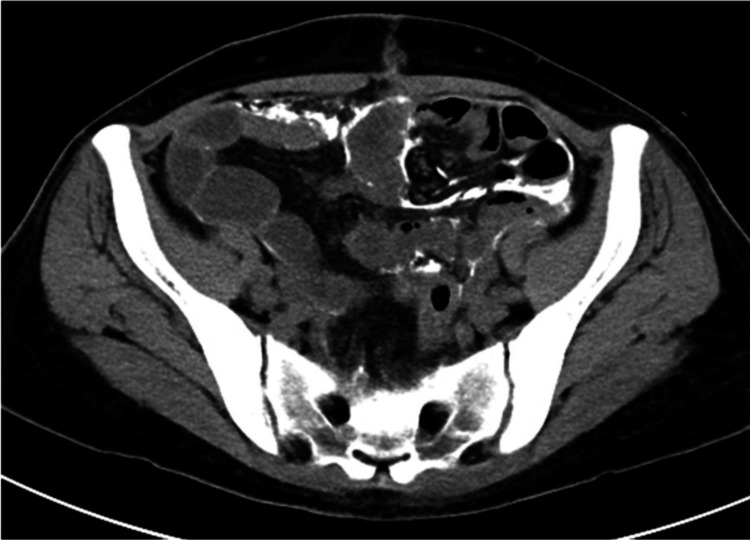
Noncontrast abdominal computed tomography performed 22 days after surgery Although barium was observed along the intestinal wall, there were no findings suggestive of intra-abdominal abscess, anastomotic leakage, or other causes of elevated inflammatory markers

**Figure 7:**
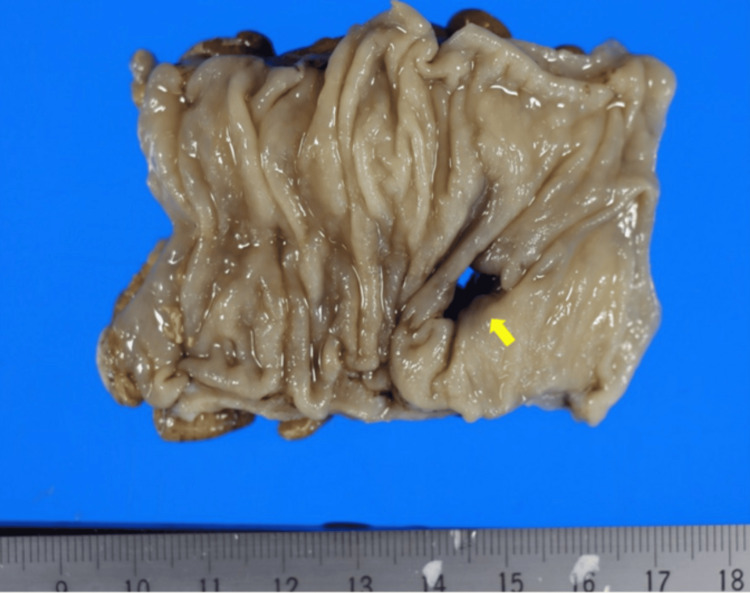
Gross findings of the resected sigmoid colon showing the perforation site (yellow arrow). No neoplastic lesion was identified. Multiple diverticula were also observed

## Discussion

Colonic diverticulosis refers to the presence of diverticula in the colon, and most cases follow an asymptomatic course. Asymptomatic colonic diverticulosis is usually detected incidentally during imaging studies or colonoscopy [12]. A previous report showed that diverticula were detected on colonoscopy in 24.7% of asymptomatic patients without a prior history of diverticulosis [13]. Meanwhile, colonic diverticulosis has been increasing worldwide [12], and similar trends have also been reported in Japan [14,15]. Therefore, opportunities for patients with asymptomatic colonic diverticulosis to undergo UGIS may further increase in the future.

Recent studies have suggested that patients with diverticulosis, including those with asymptomatic colonic diverticulosis, may show reduced colonic wall compliance, mainly characterized by reduced maximal contraction and maximal relaxation of the longitudinal muscle [16]. In addition, the diverticular colon has been reported to exhibit thickened muscle layers infiltrated by elastin, potentially contributing to abnormal motility [17]. Regardless of the presence or absence of symptoms, the presence of diverticulosis itself may be associated with abnormal bowel motility and reduced distensibility.

In this case, although a laxative had been administered after UGIS, a large amount of fecal material remained in the transverse colon. Furthermore, the perforation was identified in the sigmoid colon, and a barium fecalith was found adjacent to the perforation site. These findings suggest that, in addition to increased intraluminal pressure associated with fecal retention, reduced colonic wall compliance in the setting of asymptomatic colonic diverticulosis may have increased susceptibility to localized pressure loading caused by the barium fecalith, thereby predisposing the colon to perforation.

Although this patient was relatively young at 47 years of age, she developed postoperative septic shock and required intensive management, including temporary CHDF. Postoperatively, inflammatory markers remained elevated; however, blood cultures were negative, and CT revealed no apparent infectious source, such as pneumonia, intra-abdominal abscess, subcutaneous abscess, or anastomotic leakage. Residual barium in the peritoneal cavity or retroperitoneal space has been reported to serve as a persistent source of inflammation [5,18], suggesting that retained barium may have contributed to the prolonged inflammatory response in this case.

Although intestinal perforation after UGIS is considered a very rare complication, its actual incidence may not be fully understood. In a questionnaire survey [11], no fatal cases were identified; however, during the same period, a case was reported in which a patient died at home due to sigmoid colon perforation after a UGIS [19], and that patient also had diverticula. Furthermore, in 2024, another case of death at home after UGIS was reported, and detailed examination showed barium peritonitis due to diverticular perforation [20].

These findings suggest that severe cases may not always be reported by screening facilities and that the true incidence and clinical burden of this complication may be underestimated if assessment is limited to facilities where UGIS is performed.

Therefore, in current clinical practice, patients with previously identified colonic diverticula should be considered as potentially having reduced colonic wall compliance, regardless of the presence or absence of symptoms. When UGIS is performed in such patients, more careful postexamination evacuation management, including adequate fluid intake after the examination, administration of laxatives, and confirmation of bowel movements, may be desirable to prevent the formation of barium fecaliths. In selected cases, upper gastrointestinal endoscopy may also be considered as an alternative examination. Thus, considering not only the presence or absence of symptoms but also the presence of colonic diverticula per se may help prevent serious complications such as perforation.

## Conclusions

This was a rare but clinically important case of sigmoid colon perforation and barium peritonitis after UGIS in a patient with asymptomatic colonic diverticulosis. Although cases of barium peritonitis after UGIS have been reported previously, this case highlights the potential risk of asymptomatic colonic diverticulosis in the setting of UGIS.

Given the increasing prevalence of colonic diverticulosis, pre-UGIS risk assessment may need to consider not only a history of treated diverticulitis but also the presence of asymptomatic colonic diverticulosis. When UGIS is performed in patients with colonic diverticulosis, including asymptomatic cases, more careful postexamination bowel evacuation management may be warranted to prevent the formation and retention of barium fecaliths.
